# Labial Adhesions Causing Recurrent Urinary-Tract Infections in an Elderly Woman

**DOI:** 10.1155/2019/7584983

**Published:** 2019-12-16

**Authors:** Masashi Takemaru, Noriko Aramaki-Hattori, Chisato Tsue, Kazuo Kishi

**Affiliations:** Department of Plastic and Reconstructive Surgery, Keio University School of Medicine, Shinjuku-ku, Tokyo, Japan

## Abstract

A 91-year-old postmenopausal woman with a prior history of two labial-adhesion separations suffered from recurrent urinary-tract infections. We were able to successfully treat her labial adhesions using surgery.

## 1. Introduction

Labial adhesions are usually caused by a combination of inflammation and estrogen deficiency. Prepubertal girls and postmenopausal women are typical patients. Treatment is difficult because ointment is not effective and surgery is invasive. We report herein the case of a 91-year-old woman with labial adhesions, who suffered from dysuria and pyelonephritis.

## 2. Case Presentation

**Figure 1:**
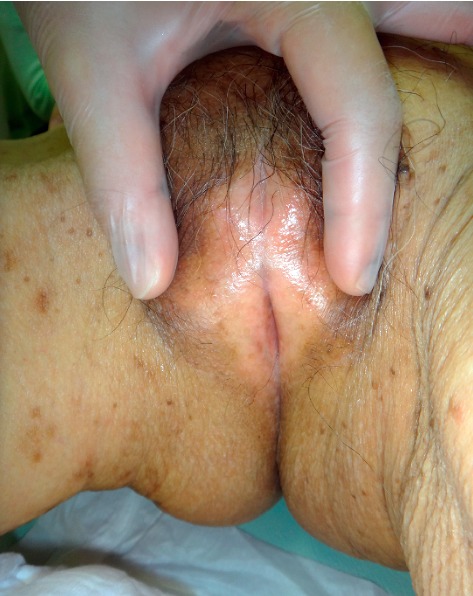
Labial fusion.

A 91-year-old postmenopausal woman with a prior history of two labial-adhesion separations suffered from recurrent urinary-tract infections. Genital examination revealed labial adhesions obscuring the external urethral orifice (Figure 1). Urinalysis showed pyuria, and her urine culture was positive for *Escherichia coli* (2 × 10^5^/mL). We prescribed topical estrogen ointment, but after 10 days the patient complained of paresthesia in her left breast. As the treatment was not having any effect at that point, we discontinued it and planned for surgical intervention under local anesthesia.

**Figure 2:**
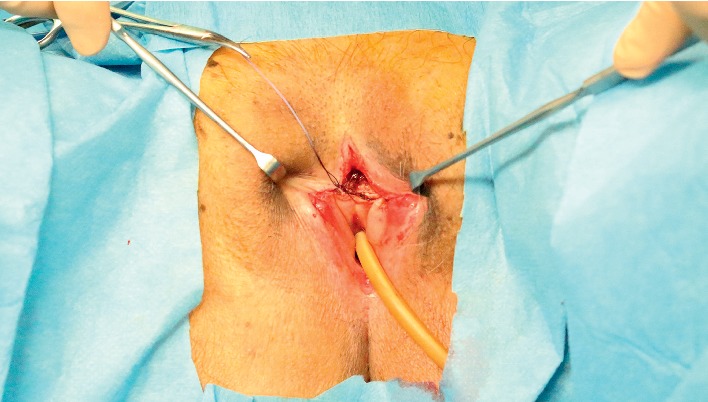
Intraoperative photography after labial separation.

**Figure 3:**
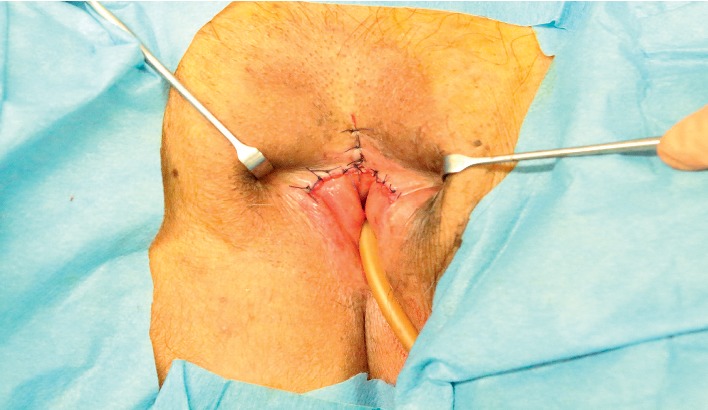
Intraoperative photography after suturing.

**Figure 4:**
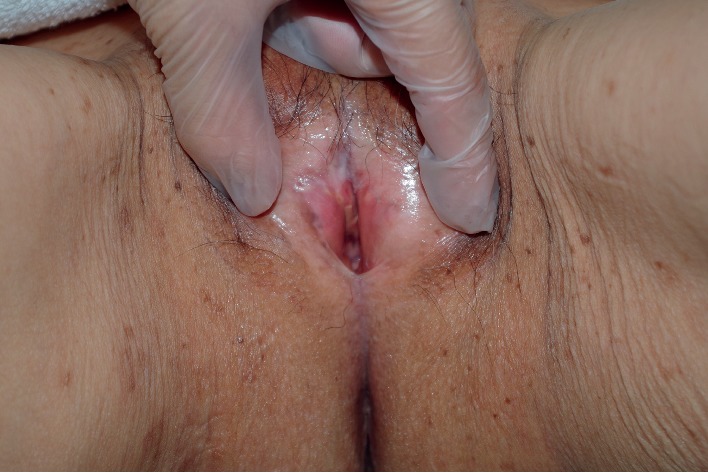
Postoperative photography after 6 months of daily petrolatum.

We separated the mucous membranes along the labial adhesion scar using a scalpel, then visually confirmed the presence of the external urethral orifice, and passed a Foley catheter to confirm its patency (Figure 2). We then used 4-0 absorbable sutures to reapproximate the cut edges of the mucous membranes, in the horizontal and vertical planes, and to restore normal spacing between the labia majora (Figure 3). Postoperatively, the patient has used petrolatum once daily to prevent readhesion. The patient has had no further urinary-tract infections in the 6 months since surgery (Figure 4).

## 3. Discussion

Labial adhesions are usually caused by a combination of inflammation and estrogen deficiency; prepubertal girls and postmenopausal women are particularly prone to this condition. While surgery improves adhesions to a greater degree than estrogen-ointment therapy, it is more invasive. For this reason, estrogen-ointment therapy is usually used in pediatric patients [1, 2]. Reportedly, 90% of patients experience resolution with a single month of therapy [3]. Estrogen ointment also prevents recurrence, although the side effects include eruption, nausea, vomiting, and breast enlargement [4–6].

When labial fusion occurs in adults and initial therapy with topical estrogen fails, surgical separation is required [7, 8]. This procedure may be performed under either general or local anesthesia. In most patients, the tissue is everted and sutured after separation of the labial adhesions. The recurrence rate is reportedly 15% [4, 6]. In our patient, we knew that the labia majora were prone to readherence because of her past history. Her recurrent urinary infections contributed to continuing inflammation, worsening the condition until the labia majora were completely adhered.

We used the Heineke–Mikulicz suturing technique, usually used in patients with pyloric stricture to provide a larger communication between the stomach and duodenum [9], to create distance between the labia majora and prevent readherence. We were able to perform this minimally invasive procedure under local anesthesia. In conclusion, our patient illustrates that surgery can be the treatment of choice in postmenopausal women with recurrent labial adhesions.
